# Insects in Classical Music

**DOI:** 10.3390/insects17090959

**Published:** 2026-09-16

**Authors:** Joseph R. Coelho

**Affiliations:** Biology Program, Quincy University, 1800 College Ave., Quincy, IL 62301, USA; j.coelho42@quincy.edu

**Keywords:** insect, music, classical, piano, symphony, butterfly, bee, fly

## Abstract

The role of insects in classical music was investigated using online sources. The prevalence of insects in classical music was much greater than anticipated; 1018 cases of insect works and pieces were discovered. Many composers repeatedly used insects in their works. The most common groups of arthropods represented were Lepidoptera, Hymenoptera, Diptera, Orthoptera and Araneae. The vast majority of cases were published recently, rather than during the traditional age of classical music from 1750 to 1830. The greatest number of composers came from the USA, followed by France, England, Austria and Germany. A number of composers of insect pieces are ranked among the best known and most productive, though most insect-themed compositions are poorly known. The music itself often evoked insects via various effects, such as buzzing, droning, and tremolo. Many insect-themed compositions were inspired by literature featuring insects. Classical music is much like other genres of music, such as rock and folk, in that it uses the same groups of species for the same reasons—such as butterflies for aesthetic value and flies for revulsion.

## Simple Summary

Insect themes in classical music were examined using internet sources. The prevalence of insects in classical music is much greater than anticipated, with over 1000 cases of insects being mentioned in the titles of musical pieces. Many composers repeatedly used insects in their works. The most common groups of arthropods represented were Lepidoptera (butterflies and moths), Hymenoptera (ants, bees and wasps), Diptera (flies and mosquitos), Orthoptera (grasshoppers), and Araneae (spiders). The use of insects in classical music is a recent phenomenon, with most published within the last one hundred years. Most composers using insects were from the USA, with the rest primarily from western Europe. Several of the composers were among the best known, though there are few familiar pieces among their insect works. The music sometimes used instrumental effects to mimic the sounds of insects. Many pieces were inspired by sources from literature, such as the fable of the ant and the grasshopper. Classical music is much like other genres of music, such as rock and folk, in that it uses the same groups of species to represent the same characteristics—such as butterflies for beauty and flies for disgust.

## 1. Introduction

Many people would quickly recognize the melody of “Flight of the Bumblebee,” but beyond Nikolai Rimsky-Korsakov’s (1844–1908) well known piece, most would be hard-pressed to name another insect-themed piece of classical music. The influence of insects on popular culture has been examined in a variety of media ranging from literature to art, cinema and beyond [1]. Recent studies on the prevalence of insect themes in music have covered rock music, folk music and music videos [2,3,4]. These works, falling under the discipline of cultural entomology, inform us about human perceptions of insects. Some taxa, especially Lepidoptera, are very popular in music because they carry positive associations. Others, such as Diptera, are mentioned frequently, but for the opposite reason. Hymenoptera may dominate because they bear both sides of the coin, having positive associations, such as honey, and negative ones, usually stinging [5]. The present study endeavours to examine the unexplored influences of insects in classical music.

Classical music is conventional music following long-established principles rather than a folk, jazz, or popular tradition. Classical music, broadly speaking, is divided into six eras: Medieval (500–1400), Renaissance (1400–1600), Baroque (1600–1750), Classical (1730–1820), Romantic (1815–1910), and Modern (1900–present) [6]. It increased in complexity and expressive depth as it evolved through these stylistic periods. Classical music in the narrow sense (4th era) may be defined as music written in the European tradition characterized by particular notation, form, and expressive elements [7,8,9]. I collected data over the full range of eras, from the year 1000 to the present. I surmised that there would be less penetration of insects into classical because popular treatments of the subject revealed only seven [10], eight [11], ten [12] and twelve [13] pieces, and mostly the same ones. This topic is an unexplored intersection of insects and human culture, as there appear to be no formal studies on the influence of arthropods in classical music.

The ways in which insects are represented in popular culture are likely to reflect how humans value them. For example, music videos, a relatively recent medium, seem to represent insects in a more positive light [14]. Classical music, being a much older genre, might be expected to demonstrate more traditionally negative views. The influence of insects in classical music will be examined through various lenses, such as the taxa represented, composer popularity, country of origin, and others.

## 2. Materials and Methods

The approach used in the study was similar to that used in previous investigations in cultural entomology, particularly insects in music [2,3,4,14,15]. Online databases were queried with a list of insect terms. Relevant search results were recorded and classified taxonomically. Results were further investigated for other relevant details, described below.

### 2.1. Data Acquisition

A list of classical music databases was developed (Table 1). Some of these databases are no longer freely available (e.g., OpenOpus), since the time of data acquisition. The searches were performed from November 2022 to February 2024. A list of insect keywords (Table 2) used to query databases was used. Four non-insect arthropod names were also included. Throughout this paper, the term insect includes all these groups. Each word, originally in English, was translated into German, French and Italian (using Google Translate), so that the primary languages used in classical music could be used to query the databases. If the keyword appeared in the title of a piece, then the composer, title, year of publication, composer’s country of origin, birth and death year were recorded if available. These raw data were starting points; each was further investigated via internet searches to fill in gaps and to confirm that the piece was, in fact, classical in style and insect-related. The determination of whether a piece is classical is necessarily subjective. This limitation is an unfortunate but unavoidable feature of this type of study, just as previous efforts have had to determine whether a song was rock and roll [2] or folk music [4]. If a piece was a movement in a larger work, then the title of that work was also recorded. Each individual piece was counted separately, regardless of whether it was part of a larger work. However, to avoid double counting, a work with an insect name was not counted if it held an insect-themed piece. When I encountered a list of a composer’s works, I examined it for additional insect content and recorded any present as data. Sometimes the same piece by the same composer (but in a different language) was discovered in different databases. Such duplicates were eliminated.

Caution was required in navigating multiple languages. It is possible that some ambiguities and errors remain, but I describe herein the ones I discovered and resolved. Using Google Translate on *Laus Deo* in Latin to English results in “Praise God.” In German, it results in “louse deodorant.” *Demoiselle* in French translates both as damsel and damselfly. There were far more damsels than damselflies in the initial data set. A piece was included only if it was reasonably clear that an odonatan damselfly was intended.

All titles were translated into English, primarily using Google Translate. This method resulted in some awkward phrasing, which was corrected when possible. Still, ambiguities persist. The German *Die Schwarze Spinne* was probably intended to mean “black widow spider,” but there was no justification to make it anything other than “black spider.”

The Ant and the Grasshopper, the fable famously told by Aesop and repeated by La Fontaine [16], was retold 22 times, primarily in French (*La Cigale et la Fourmi*); perhaps because La Fontaine was French, his book of fables was extremely popular, and this fable is first in his collection of 243. In this case, *cigale* clearly refers to a grasshopper. However, *cigale* can also be translated as cicada, making taxonomic classification of some of the pieces difficult.

In his famous *Four Seasons*, Antonio Vivaldi (1678–1741) represented buzzing gnats and flies, which do not appear in the titles, but the accompanying sonnets mention them in the Summer *Adagio e piano–Presto e forte* section. Hence, it was included. I also included some pieces that are known to have insect terms in the libretto (lyrics) but not the title. For example, George Handel’s (1685–1759) *Israel in Egypt* describes plagues of lice, flies and locusts. Similarly, Franz Schubert’s (1797–1828) “*Der Einsame*” (The Solitary), mentions crickets. It is likely that many works of this type went undiscovered, as they could not be captured by the data acquisition method. This creates a limitation in the study, but it was considered better to include the cases that were known than to exclude them.

I accepted classical music from all eras into the data. Instrumentation included orchestral music, choir, chamber music, ballet scores, operas, operettas and solo voice and instruments. Excluded were genres including children’s music, jazz, ragtime, and calypso.

I attempted to find a freely available recording of each piece, usually as a video on YouTube. The URL of each video was recorded in the data. These videos were collected into a playlist, accessible here: https://youtu.be/OGhV7fDlxxc?si=Ef3-QeOcUj7HoDcB. For each piece that was available, I listened and attempted to determine whether its musical characteristics reminded me in any way of the insect for which it was named. While this process was admittedly subjective, I believe it was still worth the attempt, as composers were likely to have intended such relationships. *Program music* is a type of instrumental music that attempts to musically render an extramusical narrative, such as the piece’s title. In contrast, *absolute music* has no narrative, i.e., it is not “about” anything [17]. The *symphonic poem*, music that illustrates a short story, was a popular form of program music during the classical period. Composers used different musical gestures to represent non-musical concepts such those from art, literature and nature [18]. I found many pieces that evoked, with reasonable clarity, images of the arthropods represented in the title.

### 2.2. Analysis

The title of each piece was examined for the types of insects mentioned. Some non-insect arthropods were also included. Spiders were assigned to Araneae, centipedes and millipedes to Myriapoda, and nonspecific terms such as “insect” were assigned to a “General” category. If more than one arthropod was mentioned in a title, each was counted. For example, “The Ant and the Grasshopper” was counted as both Hymenoptera and Orthoptera.

I attempted to determine the year of publication for each piece. It was not always possible to obtain this information, which is not surprising considering the great age of some of the compositions. Nonetheless, I was able to assign dates to over 90% of the entries. Pieces without dates were excluded from this part of the analysis, which probably biases the results toward recent work, as it was generally more difficult to find dates for the older ones. Similarly, I attempted to determine the birth and death years (vital dates) of each composer, which are provided at first mention throughout this manuscript. Some composers were active long ago when record keeping was poor. Many living composers, perhaps out of privacy, have not made their birth years public.

I attempted to determine the country of origin of each composer. Often, the composer’s country was provided by the database. If it was not, I investigated further. This effort was encumbered by the fact that countries’ names and boundaries have changed over the course of time, and many composers moved among different countries during their lives. I used the earliest country to which the composer could be assigned, unless they had moved during infancy. This process was at times subjective. I used the individual countries that once composed the USSR, and separated Czechia and Slovakia from Czechoslovakia. Less than 2% of composers could not be assigned to a country.

## 3. Results

### 3.1. General

**Figure 1:**
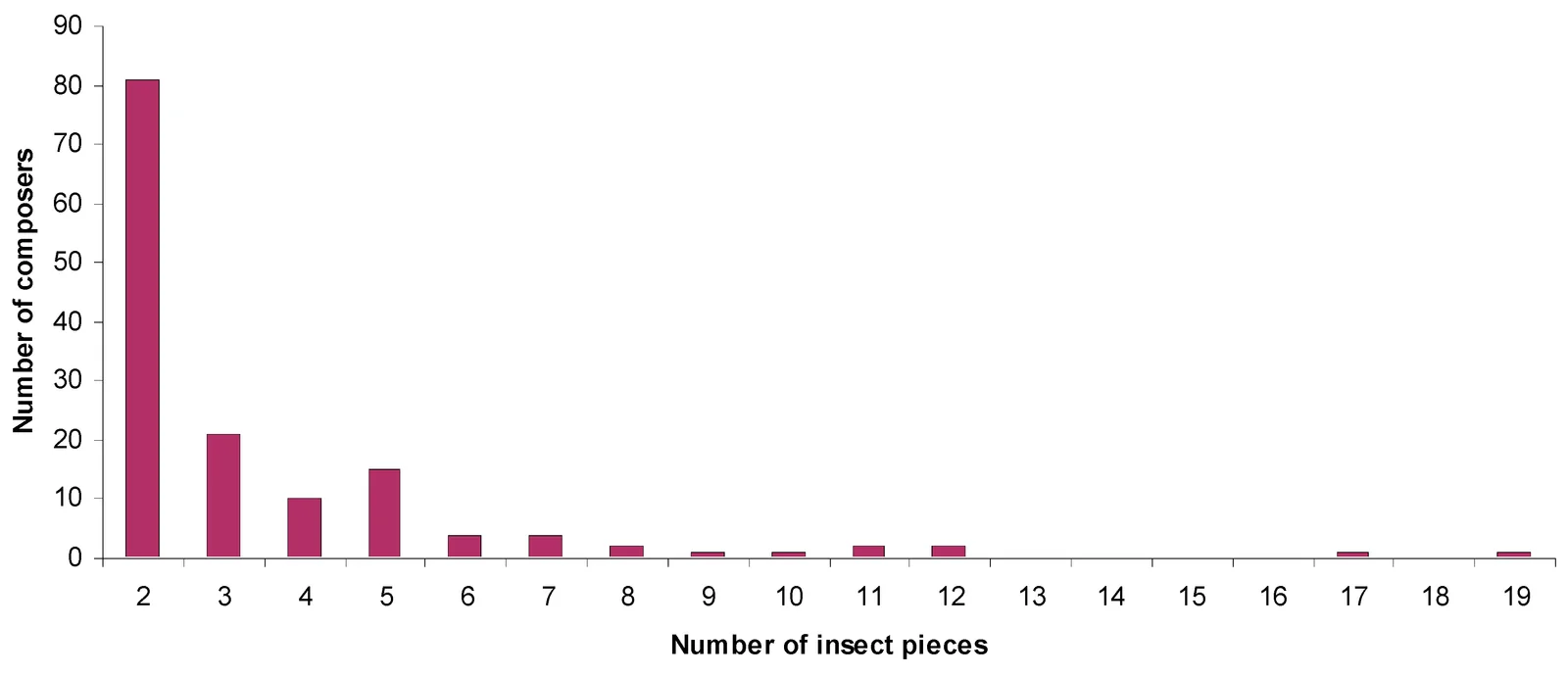
The number of composers producing two or more arthropod pieces (from a total of 649 composers).

A total of 1018 pieces of insect-themed music of the classical genre were discovered, and 649 unique composers. Half of the insect pieces were members of two or more pieces from the same composer. While two insect-themed pieces per composer were by far the most common, up to as many as five was fairly common, with a maximum of 19 (Figure 1). Multiple insect pieces as part of the same work were also common, as 58 composers produced insect-themed works with multiple insect pieces, totalling 237.

### 3.2. Taxonomy

**Figure 2:**
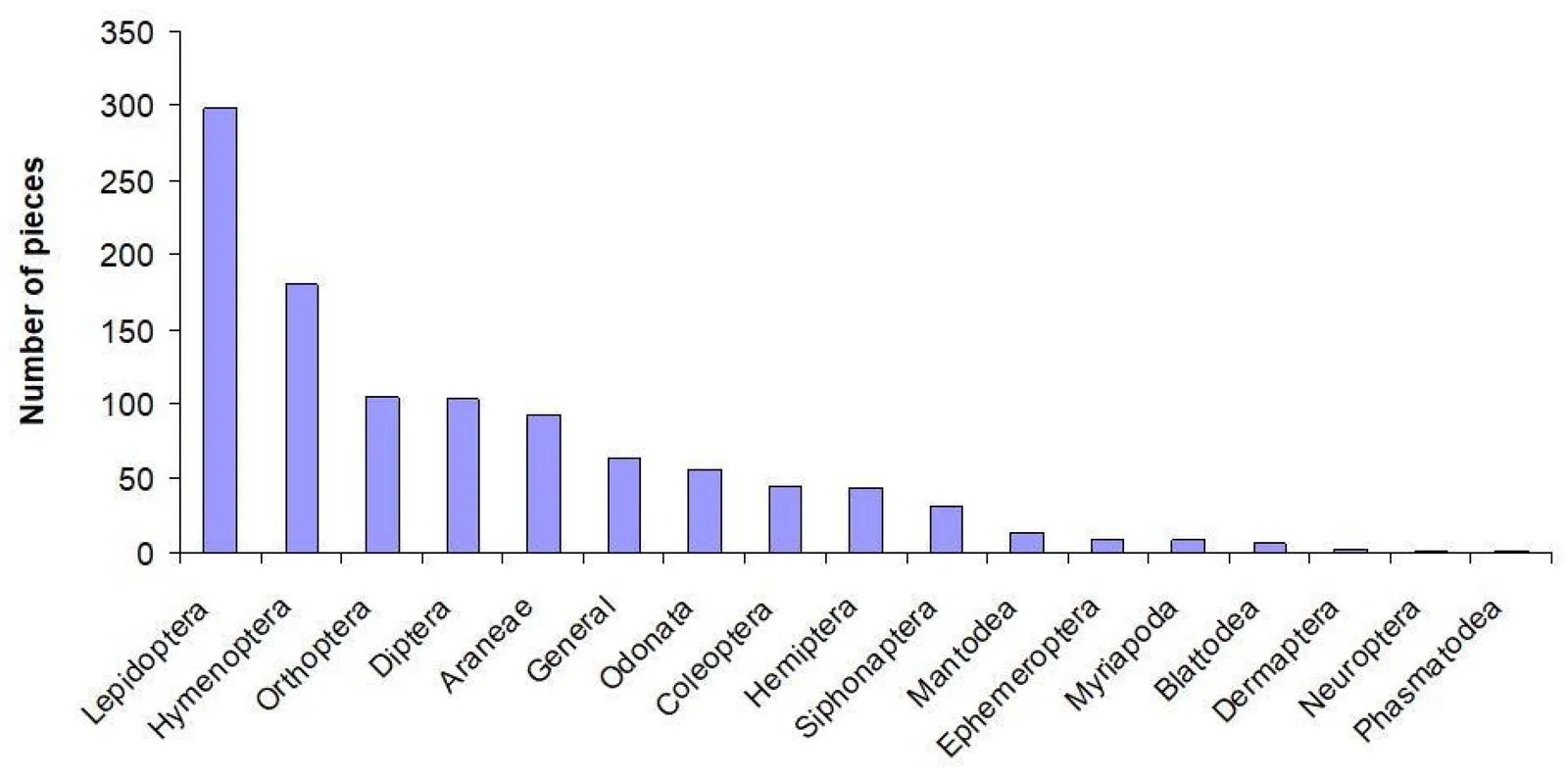
The distribution of insect-themed music among arthropods.

Among types of arthropods, Lepidoptera were by far the most common species discovered, comprising over 28% of mentions, followed by Hymenoptera. Diptera and Orthoptera tied for third most abundant, followed by Araneae (Figure 2). The general category was sixth most common, with Odonata, Coleoptera and Hemiptera providing strong showings. The rest may be considered rare, comprising less than 3% each of the total.

### 3.3. Chronology

**Figure 3:**
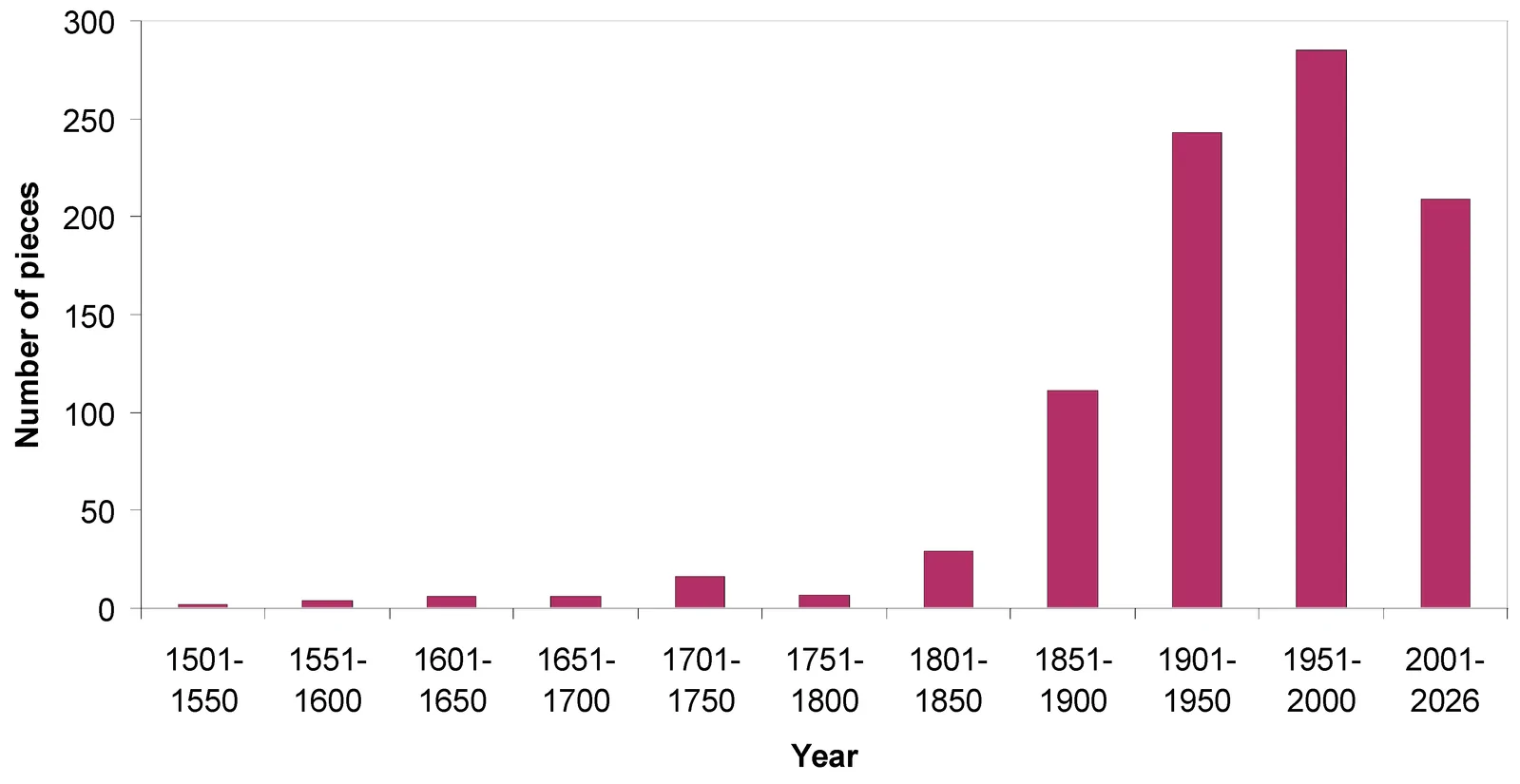
The frequency of arthropod music production over time. Only two pieces were written before 1500.

The earliest insect piece published was “Fleas,” by the poet Joseph ibn Sahl (c. 1003–1123) in 1124 [19]. Next is “El Grillo” by Josquin Des Prez (1450/1455–1521) from the 1490s. Only 22 pieces, or 1.6% of the total, were issued during the age of classical music, from 1750 to 1830. The majority of insect-related classical music was recent, over 50% being issued between the year 1950 and the present (Figure 3). There was a distinct trend of increasing frequency of insect pieces, especially during the 20th century. These patterns are essentially the same for birth years of the composers, which, therefore, are not presented here, but provided in Table S1.

### 3.4. Country

**Figure 4:**
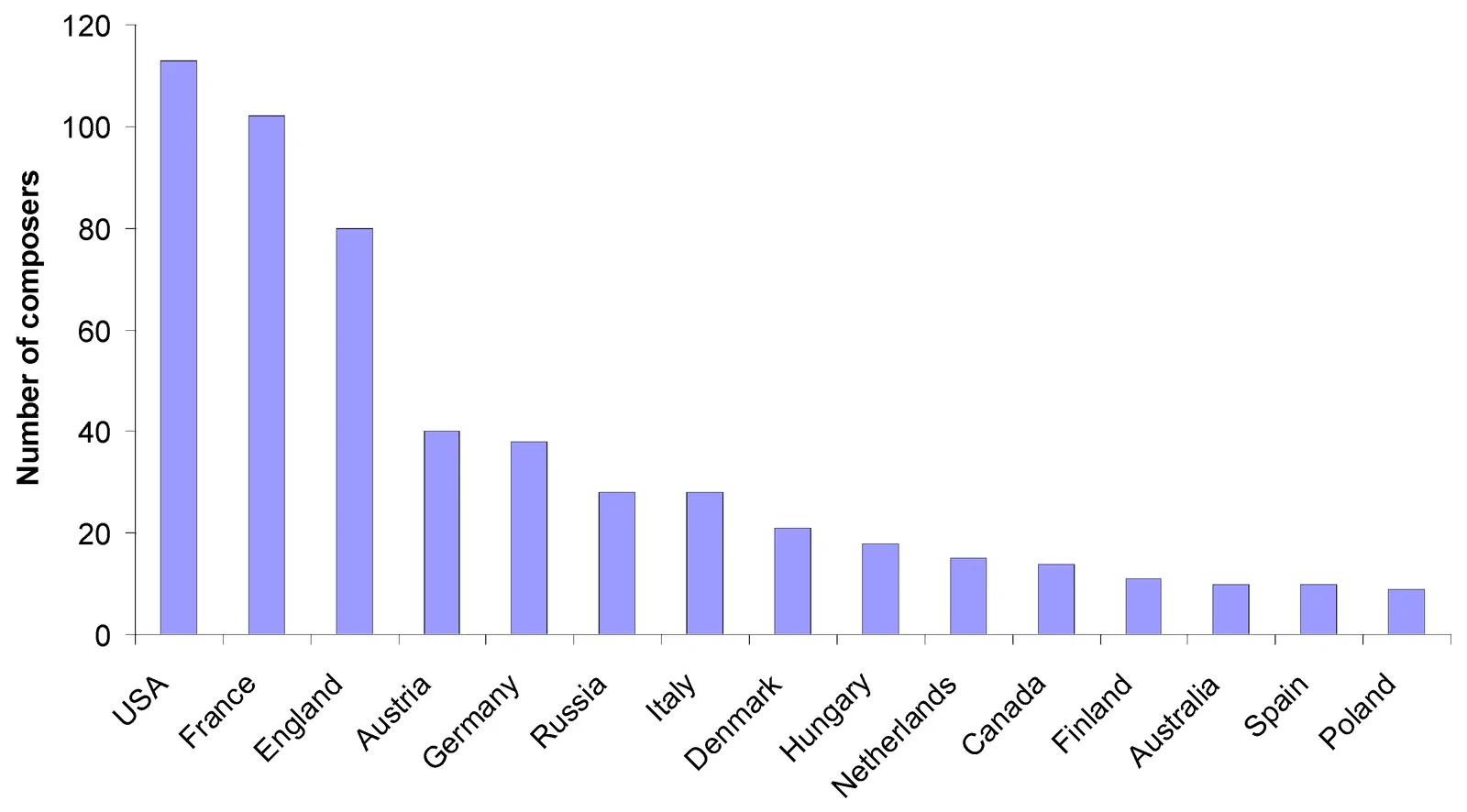
The countries of origin of arthropod music-producing composers. Those countries with fewer than three are not included.

Of 649 individual composers, the greatest number came from the USA (Figure 4). The European countries France, England, Austria and Germany followed, with Russia taking sixth place. Four more European countries were next: Italy, Denmark, Hungary and the Netherlands.

### 3.5. Music-Insect Resemblance

Forty-one pieces seemed to represent the insect(s) in the title via the sounds within (Table 3). Most common among these was an erratically rising and falling melody that evokes the irregular flight of insects such as bees and butterflies. Best known among these would be Rimsky-Korsakov’s “Flight of the Bumblebee,” which features a rapid, continuous melody built on a relentless stream of fast sixteenth notes that musically mimic the darting, erratic flight of a bee, and that makes it very difficult to play. Equally common among these were buzzing sounds, often generated with violin. Similarly, high-pitched instruments including piccolo and flute and those representing flies or mosquitoes were used. Some pieces mimicked walking, jumping or running feet, or fluttering butterflies, while others resembled actual insect calls, such as crickets stridulating or cicadas trilling. Finally, three pieces simply sounded creepy or menacing, corresponding to stereotypical perceptions of their spider subjects.

## 4. Discussion

This survey necessarily under-represents the prevalence of insects in classical music. None of the databases were truly comprehensive, and many had pieces that were not found in the others, suggesting that there are more works that were not discovered. Indeed, a few pieces suggested by friends were not found in any of them. Furthermore, pieces that may have relevant insect sounds or suggestions would not have been found if they did not have insect terms in the titles. Nonetheless, it is reasonable to expect that, with a sufficient sample size, the major trends will be robust.

Insect representation in classical music far exceeded expectations, as two to three orders of magnitude more pieces were discovered here than had previously been listed in popular sources [10,11,12,13]. Many composers seemed to have such a fascination with insects that they wrote multiple pieces about them. And these were not obscure composers. ClassicFM [20] lists the 30 greatest classical composers. Though such a list is necessarily subjective, it is interesting that 12 of these 30 wrote insect-themed music (Table 4). I list several more that are notable, and should be familiar to fans of classical music (Table 5). A similar trend was found in rock music, with the Beatles, Rolling Stones, The Who and other big names using insects in their works [2]. Insects are so ubiquitous that artists are likely to incorporate them into their music. It should be noted, however, that the insect-themed works of these composers are not necessarily their most popular works (see below).

Some lesser-known composers made interesting contributions as well. Blind French organist Louis Vierne (1870–1937) wrote “Beautiful white butterflies”, but is better known for dying at the console of the great organ of Notre-Dame on 2 June 1937, ending an otherwise powerful performance [21]. Alban Berg (1885–1935) published about 13 works, none of them insect-related, but he died from sepsis following an insect bite [22]. The list also includes some well known modern composers, such as Henry Mancini (1924–1994), known for motion picture and television soundtracks, and Peter Schickele (also known as P.D.Q. Bach, 1935–2024), a composer of comedic classical music.

The greatest producers of insect classical music were not necessarily the most famous, but simply the most prolific. A list of 30 composers from the traditional classical period and the hours of music they created [23] includes 14 (47%) that made insect-themed music (Table 6). With such an output, the range of subjects may be so great that insects were likely to be included. Unfortunately, Georg Philipp Telemann (1681–1767) does not appear on this list of 30, though he is often cited as the most productive composer in history. Beginning composing at a young age and living to 86 [24], he wrote approximately 3000 compositions [25]. Considering that half of his works were lost, his productivity in terms of hours cannot be known with certainty. But if Telemann and Johann Sebastian Bach (1685–1750), his contemporary, had the same average duration per composition (0.156 h), then Telemann’s oeuvre would comprise roughly 469 h. With such a number and duration of works, it is perhaps inevitable that some of his subjects were insects.

### 4.1. Famous Pieces

Only a few insect-themed works and individual pieces are widely popular. Vivaldi’s *The Four Seasons* is cited among one of the top 15 most famous works [26]. Certainly, Rimsky-Korsakov’s “Flight of the Bumblebee” is widely recognized. Giacomo Puccini’s (1858–1924) Opera *Madama Butterfly* deserves mention, and perhaps Hector Berlioz’s (1803–1869) “Song of the Flea.” The lack of well known pieces may be the cause of the small sample sizes in popular treatments of this topic, and the misperception that insects are rarely referenced in classical music.

Several composers were apparently so moved by insects that they composed insect-themed works containing multiple insect pieces. Those with five or more insect pieces included Isabelle Aboulker’s (1938–) *Petites Histoires Naturelles*, Kalevi Aho’s (1949–) *Hyönteissinfonia* (Insect Symphony), Leonid Bashmakov’s (1927–2016) *La Parade des Insectes*, Seymour Bernstein’s (1927–2026) *Insects Parts 1 & 2*, Roger Cichy’s (1956–) *Bugs*, Carl Davis’s (1936–2023) *A Creepy, Crawly Songbook*, Jack Curtis Dubowsky’s (1965–) *Spiders, Insects, and Bugs: Twelve Miniatures for Piano*, Jean Francaix’s (1912–1997) *L’Insectarium*, Bruce Fraser’s (1947–2017) *Carnival of the Insects*, Rued Immanuel Langgaard’s (1893–1952) *Insektarium*, Leon Louder’s (1983–) *Entomophonie*, David Rothenberg’s (1962–) *Bug Music* and *We Emerge*, Albert Roussel’s (1869–1937) *Le Festin de l’araignée*, and Franz Zebinger’s (1946–) *Schmetterlingsstücke*.

### 4.2. Taxonomy

The taxa cited by classical music (Lepidoptera, Hymenoptera and Diptera) are generally the same as those in other musical genres examined, although the order differs slightly. In rock music, Hymenoptera are most common, followed by Lepidoptera and Diptera [2]. In folk music, the order is Hymenoptera, Diptera and Lepidoptera [4]. In more visual media, such as rock videos and cover art, the order is Lepidoptera, Hymenoptera and Coleoptera [14,15].

Butterflies, moths and caterpillars seem to be the most loved among insects, as they rank high not only in insect-themed classical music, but also in rock music, folk, and music videos. Seventy-one classical pieces were named or translated as “Butterfly” or “The butterfly” or their plurals. The widespread appeal of Lepidoptera is attributed to their beauty and probably their inability to sting or bite. Hence, they almost always inspire happy and pleasant thoughts. An exception is Lori Laitman’s (1955–) “I never saw another butterfly,” which was written from the perspective of a prisoner in a concentration camp. In this case, the absence of butterflies is part of the sorrow and horror.

Hymenoptera are also frequently encountered in popular culture, and the second most common insects in classical music. The most commonly mentioned among these were bees and ants, with just a few wasps. Although there are many mentions of bees among these pieces, only three specifically mention honey bees. Many of these are attributable to the twenty-three interpretations of “The ant and the grasshopper”. Hymenoptera are unique among insects in their ability to conjure positive associations, such as the sweet honey of the bee or the strong work ethic of the ant, as well as negative associations, such as the stinging and potential aggression of wasps. These stereotypical roles make them common in rock and folk music as well.

Diptera were mostly represented by mosquitoes and generic flies. Generally, these species conjure negative images, such as Chiel Meijering’s (1954–) “The fly who bugged me” (a wordplay on *The Spy Who Loved Me*). However, many of the titles are ambiguous at best (e.g., simply “The fly”), and several have more positive titles, such as “Dance of the mosquitoes.”

Grasshoppers and crickets cannot sting, and only some of the largest can bite. They are well known to lay people and are generally well regarded. Their alleged laziness (described in “The ant and the grasshopper” accounts for a significant contribution to the data. Curiously, a genuinely negative characteristic, swarming migratory locusts, appears only twice, in Rued Immanuel Langgaard’s “*Acridium migratorium* (Migratory Locust)” and Handel’s *Israel in Egypt* as one of the Biblical plagues. Alexander Campbell Mackenzie’s (1847–1935) “The cricket on the hearth” is a nod to the classic novella by Charles Dickens [27], as are Karl Goldmark’s (1830–1915) *Das Heimchem am Herd* (1896) and Riccardo Zandonai’s (1883–1944) *Il grillo del focolare* (1908). Most orthopterans, at least the males, are able to make calls via stridulation. This characteristic is represented in several pieces, such as Gordon Getty’s (1933–) “The cricket sang.”

Fifty-seven pieces had “spider” or “spiders” in the title. Fourteen pieces had “tarantula” in the title, suggesting the outsized impact of large mygalomorph spiders on the public. Ten spider-themed pieces were named *tarantella*, an Italian dance which was thought to result from the bite of a tarantula spider or to serve as its cure. Nine pieces mentioned “black widow,” “black spider” or “red-backed spider,” referencing the well known, widespread, venomous genus *Latrodectus*. The negative characteristics of spiders seem to be getting most of the attention here, with a few exceptions, such as the *tarantella* cure.

### 4.3. Chronology and Country

Very little insect-themed music was produced during the golden age of classical music, from 1750 to 1830, and almost none before that. The old masters focused on topics primarily related to religion and, secondarily, romance. However, the conclusion that the inclusion of insects in the classical genre is primarily a recent phenomenon must be tempered with the knowledge that this effect partly reflects better preservation and indexing of recent works as well as an increase in the volume of music produced over time. It may also be related to the fact that most of the composers are from the USA. The average year of publication for an insect piece from a composer in the USA is 1974, while for France it is 1879, England 1907 and Austria 1920. The country of origin and the timing of composition are related.

### 4.4. Insect/Music Resemblances

A modest number of pieces invoked the sound of real insects in various ways. Rimsky-Korsakov’s “Flight of the bumblebee,” published in 1900, is the best known among these. Written in a blistering fast tempo (often around 144 to 167 beats per minute), it requires extreme technical agility from the performer. The melody relies heavily on chromatic scales—moving by half-steps—which creates a sonic texture, rather than a traditional lyrical tune. Set in the key of A minor, the piece has a tense, urgent, and restless atmosphere. The notes dart rapidly up and down in pitch, capturing sudden shifts in direction similar to a bee in flight. Curiously, it is predated by Antonio Pasculli’s (1842–1924) 1874 “*Le api* (The Bees),” which uses a similar contour of up and down pitch. This pattern is relatively common, and a simple way of mimicking insects with music.

Ralph Vaughan Williams’s (1872–1958) “The Wasps (Overture),” features a loud, raspy, and energetic musical imitation of swarming wasps. It opens the piece with a sudden, aggressive sequence of buzzes that lasts for about two minutes before transitioning into traditional English folk-style melodies. The buzzing effect is created by specific orchestral techniques that mimic the rapid fluttering of wings, such as trills in the upper strings and woodwinds, fast, darting scale passages running up and down, sudden loud accents that mimic stinging or jabbing movements, and dynamic swells and orchestral stabs. The frantic buzzing serves as a theatrical curtain-raiser for Aristophanes’s classical Greek comedy, representing the chorus of old jurymen in the play who are dressed as wasps.

One musical method inspired by buzzing insects is a *drone*—a harmonic effect in which a single note or a chord is sounded continuously throughout a piece [28]. One common instrument used to emulate insects via a drone is the violin, which can produce a buzz by tremolo, or the rapid repetition of the same note. Overall, however, the instruments used were more diverse, including piano, saxophone, trumpet and accordion. In some cases, different instruments were used to represent the same insect. In Josquin Des Prez’s (1450–1455) “*El grillo* (The Cricket),” chattering vocal runs mimic crickets. Ferde Grofé (1892–1972), however, used trumpets to sound like crickets [29]. George Enescu’s (1881–1955) *Grillon* (Cricket) has squeaky cricket noises at the beginning made by violin and piano. Franz Schubert’s *Der Einsame* (The Solitary) features a piano baseline that resembles crickets chirping, while crickets are also mentioned in the lyrics [30]. David Diamond’s (1915–2005) “Scherzo: Insects around the flame” is an instrumental work written for flute and piano, representing an insect’s frantic death-dance, flitting around a candle flame.

Joseph-Bodin de Boismortier’s (1689–1755) “*La puce* (The flea)” is played on the harpsichord, which mimics the jumping of fleas in part by using a similar mechanism of action. In a flea’s legs, slow muscle contraction stores elastic energy in the elastomeric protein resilin. The stored energy is suddenly released to launch the flea in a jump [31]. A harpsichord creates sound by plucking strings with small, plectrum-tipped levers called jacks, rather than striking strings with hammers as in the piano. When a key is pressed, its jack rises, the plectrum stretches the string, storing elastic energy until the string slips past the end of the plectrum, allowing the string to recoil and vibrate, making a note [32]. The ukulele was named by the Hawaiians, translating as “jumping flea,” perhaps for similar reasons, though other etymological explanations dominate [33].

### 4.5. Program Music

Composers of classical music rarely write their own lyrics. They either borrow from existing works or enlist another person to be the lyricist. Program music was common, as many pieces were based on poems or literature. As mentioned, the frequency of “The ant and the grasshopper” is a result of La Fontaine’s fable, generating 22 interpretations. Similarly, “Where the bee sucks” from Shakespeare’s *The Tempest* [34] is represented by seven cases. “The spider and the fly” entitles five pieces, a tribute to the famous cautionary poem from Mary Howitt [35]. *The Cricket on the Hearth: A Fairy Tale of Home* is a novella by Dickens [27], and is represented by three examples. “The butterfly that stamped” is from *Just So Stories* by Kipling [36], and two pieces were written to represent it. Arthur Farwell’s (1872–1952) “The level bee,” one of *12 Emily Dickinson Songs, Op. 105*, is based on *The Bee* [37], the second line of which is “I hear the level bee.” Hence, it appears that many insect-themed musical pieces were inspired by popular literature referencing insects.

The sensational themes that appear frequently in rock music, such as giant insects, chimeric human–insects and large swarms [2,3,14,15] are absent from classical music. However, classical music is not as serious and staid as most believe. Bawdy songs are well known, especially from the earliest pieces, and a collection of 33 are even available as a CD [38].

## 5. Conclusions

In summary, insects were included in classical music much more often than anticipated, and were invoked by even some of the most revered composers. Some composers used them repeatedly in their works. However, only a few of these pieces remain popular today. The kinds of arthropods most often mentioned, such as butterflies, bees and ants, are the same as those frequently used in other media and genres of music. Most insect-themed music was produced relatively recently, and by composers in the USA and western Europe. Insects are often characters in stories that are put to music. While it may not be possible to assess whether insects are regarded in a negative or positive way, there seems to be not much difference from other genres of music in this regard. Classical composers use the same taxa, and explore similar themes.

## Figures and Tables

**Table 1:** Online databases queried for insect terms.

Name	URL
All Music	allmusic.com (accessed on 10 September 2026)
Arkiv Music	https://arkivmusic.com/pages/classical-home (accessed on 10 September 2026)
Classical Net	http://www.classical.net/music/links/database.php (accessed on 10 September 2026)
Classical Archives	https://www.classicalarchives.com/newca/#!/ (accessed on 10 September 2026)
Dictionary of American Classical Composers	https://www.google.com/books/edition/Dictionary_of_American_Classical_Compose/WR8jAQAAQBAJ (accessed on 10 September 2026)
Henry’s records	https://www.henrysrecords.org/ (accessed on 10 September 2026)
Music and Musicians	https://www.musiqueorguequebec.ca/ (accessed on 10 September 2026)
Music Brainz	musicbrainz.org (accessed on 10 September 2026)
Music Web International	musicweb-international.com (accessed on 10 September 2026)
Musicalics	https://musicalics.com/en (accessed on 10 September 2026)
Naxos Works Database	https://www.naxos.com/feature/Naxos-Works-Database.asp (accessed on 10 September 2026)
Online Archive of California	https://oac.cdlib.org/ (accessed on 10 September 2026)
Open Opus	https://openopus.org/ (accessed on 15 November 2022)
Sheet Music Plus	sheetmusicplus.com (accessed on 10 September 2026)
Song of America	https://songofamerica.net/ (accessed on 10 September 2026)
Subito Music Corporation	https://www.subitomusic.com/ (accessed on 10 September 2026)
Vassar	https://library.vassar.edu/az.php?s=48527 (accessed on 10 September 2026)

**Table 2:** List of search terms used to find insect-themed music.

Taxon	English	German	Italian	French
Myriapoda				
	millipede	Tausendfüßler	millepiedi	mille-pattes
	centipede	Tausendfüßler	centopiedi	scolopendre
Araneae				
	tarantula	Tarentule	tarantola	tarentule
	spider	Spinne	ragno	araignée
Ephemeroptera				
	mayfly	Eintagsfliege	effimera	éphémère
Odonata				
	dragonfly	Libelle	libellula	libellule
	damselfly	Jungfer	damigella	demoiselle
Dermaptera				
	earwig	Ohrwurm	forbicina	perce-oreille
Orthoptera				
	grasshopper	Heuschrecke	cavalletto	sauterelle
	cricket	Kricket	cricket	criquet
	katydid	Katydid	catadide	katydide
Phasmatodea				
	walkingstick	Stabheuschrecke	insetto stecco	phasme
Mantodea				
	mantis	Gottesanbeterin	mantide	mante
Blattodea				
	cockroach	Kakerlake	scarafaggio	cafard
Phthiraptera				
	louse	Laus	pidocchio	pou
Hemiptera				
	cicada	Zikade	cicala	cigale
Coleoptera				
	beetle	Käfer	scarafaggio	scarabée
	weevil	Rüsselkäfer	tonchio	charançon
	ladybug/ladybird	Marienkäfer	coccinella	coccinelle
	dung beetle	Mistkäfer	Scarabeo stercorario	bousier
Diptera				
	fly	Fliege	mosca	mouche
	maggot	Made	verme	asticot
	mosquito	Moskito	zanzara	moustique
Siphonaptera				
	flea	Floh	pulce	puce
Lepidoptera				
	caterpillar	Raupe	bruco	chenille
	moth	Motte	falena	papillon
	butterfly	Schmetterling	farfalla	papillon
Trichoptera				
	caddisfly	Köcherfliege	caddisfly	phrygane
Hymenoptera				
	ant	Ameise	formica	fourmi
	bee	Biene	Ape	abeille
	wasp	Wespe	vespa	guêpe
General				
	insect	Insecte	insetto	insekt
	bug	Bogue	insetto	insekt

**Table 3:** Number of insect-themed pieces that bear sounds resembling insect characteristics. For specific pieces, see Table S1.

Insect Trait	Number of Pieces
Erratic flight	11
Buzzing	11
High-pitched fly	7
Footfalls	7
Fluttering	4
Suspense, horror	3
Insect calls	3

**Table 4:** The greatest classical composers, ranked according to ClassicFM [20], and their vital dates (year of birth and death). Those who published insect-themed music are shown in red.

Rank	Name	Vital Dates
1	Mozart, Wolfgang Amadeus	1756–1791
2	Bach, Johann Sebastian	1685–1750
3	Beethoven, Ludwig van	1770–1827
4	von Bingen, Hildegard	1098–1179
5	Monteverdi, Claudio	1567–1643
6	Handel, George Frideric	1685–1759
7	Vivaldi, Antonio	1678–1741
8	Debussy, Claude	1862–1918
9	Tchaikovsky, Pyotr Ilyich	1840–1893
10	Haydn, Joseph	1732–1809
11	Schumann, Robert	1810–1856
12	Elgar, Edward	1857–1934
13	Verdi, Giuseppe	1813–1901
14	Wagner, Richard	1813–1883
15	Strauss, Richard	1864–1949
16	Mahler, Gustav	1860–1911
17	Schubert, Franz	1797–1828
18	Saint-Saens, Camille	1835–1921
19	Stravinsky, Igor	1882–1971
20	Chopin, Frédéric	1810–1849
21	Williams, Ralph Vaughan	1872–1958
22	Beach, Amy Marcy	1867–1944
23	Mendelssohn, Felix	1809–1847
24	Shostakovich, Dmitri Dmitrievich	1906–1975
25	Brahms, Johannes	1833–1897
26	Dvořák, Antonin	1841–1904
27	Rachmaninov, Sergei	1873–1943
28	Glass, Philip	1937–
29	Bernstein, Leonard	1918–1990
30	Williams, John	1932–

**Table 5:** Famous classical composers who published insect music, unranked.

Name	Vital Dates
Bartok, Bela	1881–1945
Bizet, Georges	1838–1875
Couperin, François	1668–1733
Debussy, Claude	1862–1918
Mancini, Henry	1924–1994
Mozart, Franz Xavier (Wolfgang Amadeus, Jr.)	1791–1844
Mussorgsky, Modest Petrovich	1839–1881
Prokofiev, Sergei	1891–1953
Puccini, Giacomo	1858–1924
Purcell, Henry	1659–1695
Schickele, Peter (P.D.Q. Bach)	1935–2024
Telemann, Georg Philipp	1681–1767

**Table 6:** The rank of composers from the traditional era in terms of their musical output (from [3]). Names of composers of insect-themed music are shown in red.

Rank	Composer	Hours
1	Mozart	240
2	Vivaldi	235
3	Haydn	180
4	Bach	175
5	Liszt (Piano)	121
6	Beethoven	96
7	Schubert	82.8
8	Britten	78
9	Richard Strauss	76.8
10	Tchaikovsky	66
11	Wagner	62.4
12	Shostakovich	58.8
13	Brahms	55.2
14	Dvorak	54
15	Schumann	54
16	Sullivan	45
17	Bartok	38.4
18	Rachmaninoff	38.4
19	Stravinsky	36
20	Walton	27.6
21	Poulenc	24
22	Debussy	21.6
23	Scriabin	21.6
24	Crumb	20.4
25	Mahler	19
26	Chopin	18
27	Ravel	16
28	Boulez	14
29	Ligeti	12
30	Satie	12

## Data Availability

All data used in this study are available in the Supplementary Material or readily found on the internet.

## Supplementary Materials

The following supporting information can be downloaded at: https://www.mdpi.com/article/10.3390/insects17090959/s1, A spreadsheet containing all of the data used in the study accompanies the manuscript: Table S1: Composers of insect-themed classical music, their vital dates, pieces and works, year of issue, composer’s country of origin, taxa represented, resemblance to insects and links to videos.
